# Dihydromyricetin: an emerging compound with comprehensive effects on multiple systems

**DOI:** 10.3389/fphar.2024.1488003

**Published:** 2025-01-03

**Authors:** Chengyi He, Yunfei Chen, Jiao Xie, Miao Luo, David Fisher, Nguyen Thi Thu Hien, Erkin Musabaev, Yiping Dang, Lei Zhao, Yin Xia

**Affiliations:** ^1^ College of Integrative Medicine, Fujian University of Traditional Chinese Medicine, Fuzhou, China; ^2^ Department of Vascular Surgery, Union Hospital, Tongji Medical College, Huazhong University of Science and Technology, Wuhan, China; ^3^ Health Management Center, Union Hospital, Tongji Medical College, Huazhong University of Science and Technology, Wuhan, China; ^4^ Department of Infectious Diseases, Union Hospital, Tongji Medical College, Huazhong University of Science and Technology, Wuhan, China; ^5^ Department of Medical Biosciences, Faculty of Natural Sciences, University of The Western Cape, Cape Town, South Africa; ^6^ Hai Phong University of Medicine and Pharmacy, Haiphong, Vietnam; ^7^ The Research Institute of Virology, Ministry of Health, Tashkent, Uzbekistan; ^8^ Department of Vascular Surgery, The Affiliated People’s Hospital of Fujian University of Traditional Chinese Medicine, Fuzhou, China

**Keywords:** dihydromyricetin, anti-inflammatory, antioxidant, anti-virus, signaling pathway

## Abstract

Dihydromyricetin (DHM or DMY) is a flavonoid derived from natural sources with a range of confirmed biological benefits. It exhibits anti-inflammatory, antioxidant, anti-tumor, and anti-viral activities. DHM is recognized for its high biosafety, making it a promising subject for further research. This article offers a comprehensive overview of DHM’s pharmacological properties, mechanisms, and recent research developments in the cardiovascular, urinary, digestive, nervous, and respiratory systems. The review summarizes DHM’s biological effects and associated signaling pathways, providing novel insights for its clinical application.

## Graphical Abstract

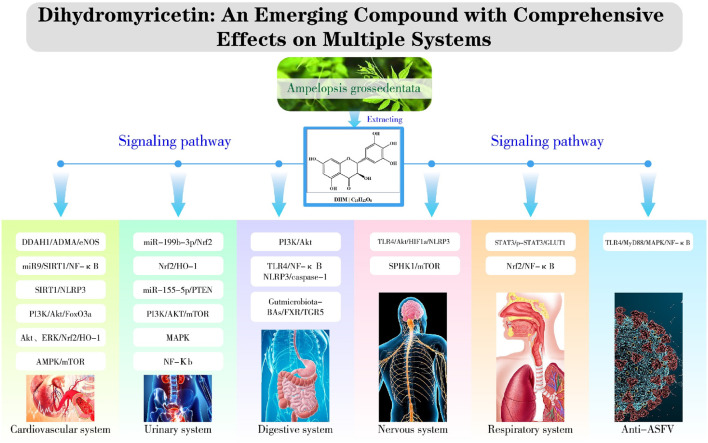

## Highlights

• Dihydromyricetin is a promising natural product with a high safety profile and broad biological activity.
• The main pharmacological effects of DHM are anti-inflammatory, antioxidant, antiviral, anti-tumor and metabolic regulation.
• A review of DHM, including its potential mechanisms of action on different systems in the human body and related signaling pathways.
• Discussing the main factors affecting the development and utilization of dihydromyricetin, including its stability and relatively low bioavailability. Discussing how to improve its bioavailability.

## 1 Introduction

**FIGURE 1:**
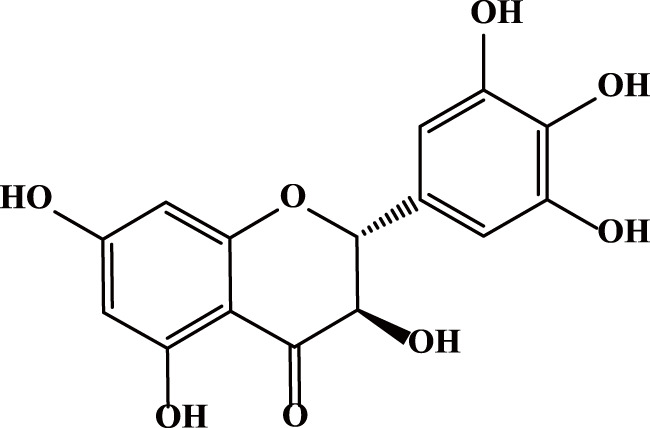
The chemical structure of DHM.

Dihydromyricetin (DHM or DMY) is a flavonoid extracted from the young stems and leaves of Ampelopsis grossedentata. It is a polyphenolic hydroxy dihydroflavanol with a molecular weight of 320.25 g/mol and a molecular formula of C_15_H_12_O_8_ (Figure 1) (Guan et al., 2019). DHM is widely distributed in plants such as grapes, mulberries, and ginkgo biloba. Particularly high concentrations are found in vine tea (Liu D et al., 2019), reaching up to 30%–40%. It has been demonstrated to possess multiple pharmacological activities, such as anti-inflammatory, antioxidant, and anti-tumor effects (Zhang et al., 2018). Notably, it is nearly non-toxic and demonstrates an excellent safety profile (Semwal et al., 2016). The toxicity of DHM has been found to be very low, with previous acute toxicity tests showing that the safe dose of DHM in rats is 10 g/kg (Xu et al., 2008). Using the body surface area normalization method, the estimated maximum safe dose for mice is around 16 g/kg, and for adults it may be 1.6 g/kg (Zeng et al., 2023).

The phenolic hydroxyl groups in DHM predominantly contribute to its chemical instability, which is influenced by pH buffers and metal ions such as Fe³⁺, Al³⁺, and Cu^2^⁺ (Xiang et al., 2017), DHM exhibits stability in weakly acidic environments but becomes unstable under alkaline conditions (Liu et al., 2019). Additionally, temperature significantly affects DHM’s stability; for instance, the concentration of free DHM in a solution of 60 μg/mL decreased by 40% when exposed to 60°C for 16 days (Liu et al., 2012).

## 2 Pharmacological actions of DHM

**FIGURE 2:**
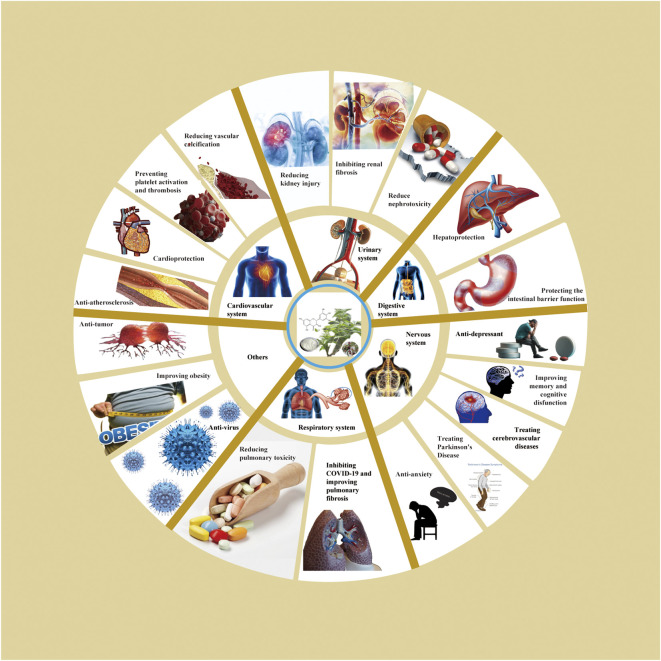
Pharmacological effects of DHM.

**FIGURE 3:**
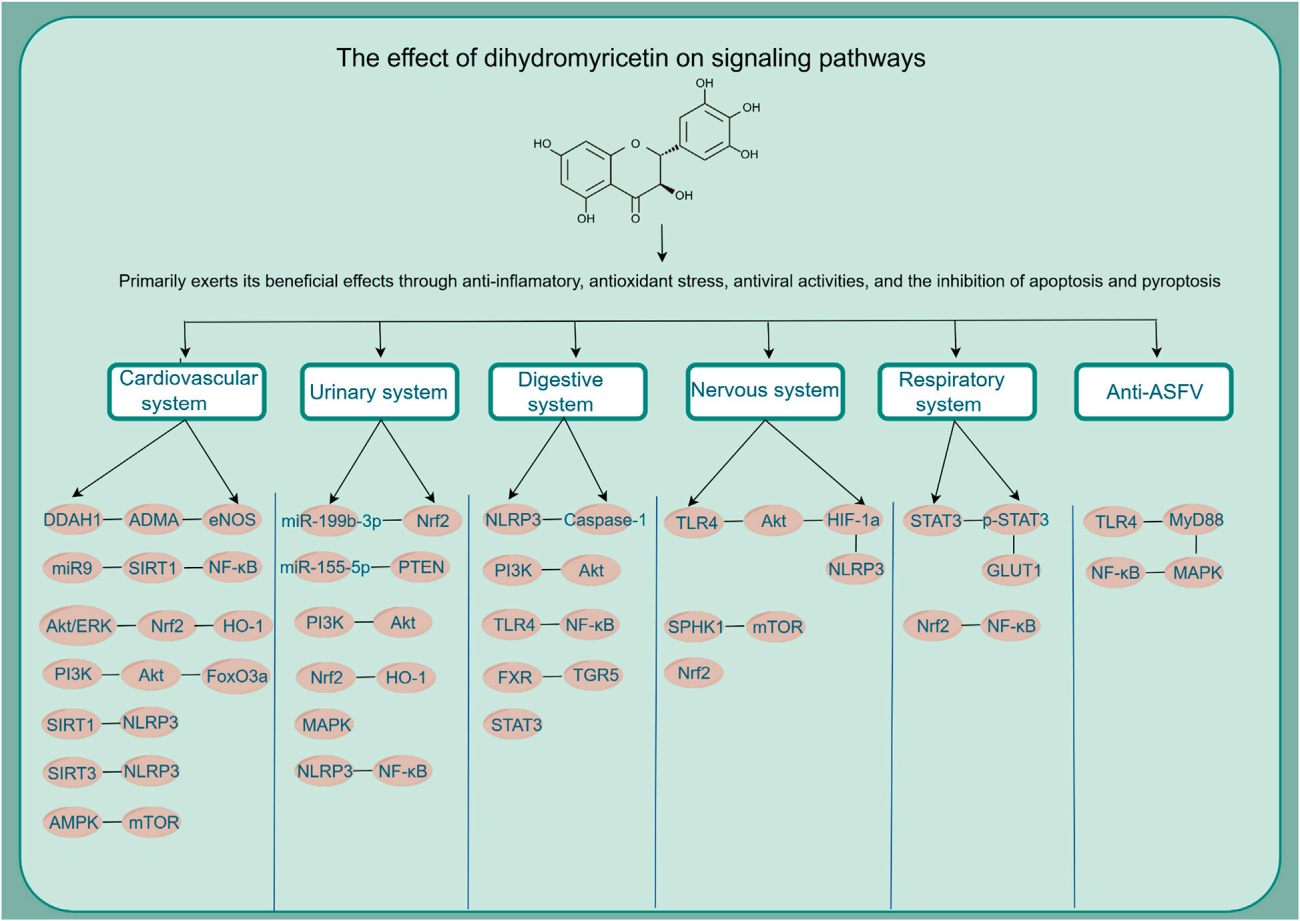
The effect of dihydromyricetin on signaling pathways.

Based on relevant cellular and animal studies, DHM has demonstrated a diverse array of pharmacological properties, including antioxidant, anti-inflammatory, anti-tumor, and anti-viral effects. Given its exemplary safety profile, DHM shows substantial potential for clinical applications. Recent research has allowed us to compile a summary of DHM’s pharmacological impacts on various organs and systems, as illustrated in Figure 2. Additionally, we have detailed the different signaling pathways influenced by DHM in Table 1 and Figure 3.

**TABLE 1:** DHM exerts beneficial effects through multiple signaling pathways.

	Disease/Target	Experimental model/materials	Mechanism	Signaling pathway	Ref
DHM	Atherosclerosis	HCD mice	DHM→miR21↓→DDAH1/ADMA/eNOS/NO↑	DDAH1/ADMA/eNOS	Yang et al. (2020)
		HCD mice; LPS + INF-γ-induced BMDMs	DHM→M1 markers (IL-1β、Tnf-α、IL-6、Nos2)↓, M2 markers (IL-10、Arg1)↑ →miR9↓→ SIRT1↑, NF-κB↓→M1 macrophage polarization↓	miR9/SIRT1/NF-κB	Yang et al. (2023)
		Ox-LDL-induced HUVEC	DHM→restores mitochondrial membrane potential →ROS、MAD↓, SOD、CAT、GSH-Px↑→oxidative stress↓ →caspase-3、caspase-9、cytochrome C↓, Bcl-2/Bax↑→apoptosis↓ →Akt、ERK↑→Nrf2/HO-1↑	Akt、ERK/Nrf2/HO-1	Luo et al. (2017)
		SNP-induced HUVEC	DHM→MAD↓, SOD↑→oxidative stress、apoptosis↓ →p-Akt、p-FoxO3a↑→oxidative stress↓	PI3K/Akt/FoxO3a	Zhang et al. (2019)
	DCM	SIRT3-KO mice; Ox-LDL-induced Macrophages	DHM→SIRT3↑→cellular cholesterol↓, foam cell formation↓ →SIRT3↑→NLRP3↓, improves mitochondrial function	SIRT3/NLRP3	Ding et al. (2021)
		Diabetic mice	DHM→miR-34a↓→restoring damaged autophagy, mitigating cardiac dysfunction	NA	Ni et al. (2020)
		Diabetic mice; SIRT3-KO mice	DHM→FBG、TG、HbA1c↓, FINS↑ →EF、FS、E/A↑→Improving Cardiac Dysfunction →SIRT3↑, NLRP3、IL-1β、caspase 1↓	NA	Chen et al. (2023a)
	Cardiotoxicity	Doxorubicin-induced rats; Doxorubicin-induced H9C2 cell	DHM→LVEF、LVFS↑, LVIDd、LVIDs↓→attenuating left ventricle dysfunction →Bax/Bcl-3↓→ attenuating cardiac apoptosis →SIRT1↑→NLRP3、caspase-1、IL-1β、IL-18↓	SIRT1/NLRP3	Sun et al. (2020)
		Doxorubicin-induced mice; Doxorubicin-induced AC16 cell	DHM→LVEF、LVFS↑, LVIDd、LVIDs↓→attenuating left ventricle dysfunction →ROS in AC16 cell↓→oxidative stress↓ →Bax、Bcl2、cleaved caspase-3↓→ameliorating apoptosis response →p-AMPK、Beclin-1、LC3-II↑, mTOR active↓→protective autophagy↑	AMPK/mTOR	Li X et al. (2022)
	Anti-thrombotic	Washed human platelets; Thrombin-stimulated HUVECs; Ferric chloride-induced mice	DHM→P-selectin、platelet integrin αIIbβ3、Ca^2+^↓→platelet activation and adhesion *in vitro*↓ →vWF、PDI↓→endothelial activation↓ →fibrin deposition↓→ thrombus formation *in vivo*↓	NA	Chen et al. (2021)
	AKI	LPS-induced AKI rats	DHM→KIM1↓、BUN↓	NA	Wang et al. (2016)
		CLP-induced Septic AKI mice	DHM→Nrf1、HO-1、NQO-1↑, IL-6、TNF-a、KIM-1、miR-199b-3p↓	miR-199b-3p/Nrf2	Tian et al. (2021)
DHM	Renal Fibrosis	*miR-34a* ^*−/−*^ mice; UUO mice	miR-34a Deficiency→Ameliorates Renal Fibrosis DHM→miR-34a↓→Klotho↑→Inhibit renal fibrosis	NA	Liu Y et al. (2019)
		DN rat; HG induced NRK-52E cell	DHM→miR-5-52p↓, PTEN↑ →p-PI3K、p-AKT、p-mTOR↓	miR-155-5p/PTEN PI3K/Akt/mTOR	Guo CH et al. (2020)
	Nephrotoxicity	CS-DHM-NPs; Cisplatin induced AKI mice	DHM→Nrf2↑, SOD、CAT↑, IL-6、IL-1β、TNF-α↓	Nrf2	Yan et al. (2023)
		Cisplatin induced AKI mice; Cisplatin induced HK-2 cells	DHM→Nrf2、SOD、CAT↑,HO-1、GCLC、GCLM、p62↑ →p-ERK、p-JNK↓ →NF-κB、NLRP3↓	Nrf2/HO-1 MAPK NLRP3/NF-Κb	Xu et al. (2023)
	CLI	CCl_4_ induced CLI mice	DHM→NLRP3、IL-1β、caspase-1、GSDMD-N↓→reducing pyroptosis	NLRP3	Cheng et al. (2020)
	Hepatic Injury	LPS-induced hepatic injury chickens	DHM→SOD、GSH-Px↑, MDA、H2O2↓→inhibiting oxidative stress →NLRP3、caspase-1↓ →Gasdermin A、IL-1β、IL-18↓→inhibiting pyroptosis	NLRP3	Shi et al. (2022)
	Liver Fibrosis	TTA-induced liver fibrosis mice	DHM→TGF-β1、α-SMA↓ →PI3K、AKT↓, Bcl-2、Bcl-XL↑, Bax、cleaved Caspase-9、cleaved Caspase-3、NF-κB、TNF-α、IL-1β↓	PI3K/Akt	Zhao et al. (2021)
	Hepatotoxicity	MTX-induced hepatotoxic rats	DHM→TLR4、NK-κB p65↓ →NLRP3、caspase-1、IL-1β、IL-18↓	TLR4/NF-κΒ NLRP3/caspase-1	Matouk et al. (2022)
	IBD	DSS-induced colitis in mice	DHM→Lactobaccillus and Akkermansia in colitis↑ →LCA、CDCA↑, TGR5、FXR↑	FXR/TGR5	Dong et al. (2021)
	Intestinal Barrier Destruction	HFD-Induced Intestinal Barrier Destruction mice	DHM→STAT3 activation↑→p-ERK、p-CREB↑→IL-22↑	STAT3	Zhou et al. (2023)
	Depression	LPS induced neurotoxic mice	DHM→TLR4、CD_14_、NF-κB p65、p-NF-κB p65↓ →PDPK1、p-Akt、p-GSK-3β、HIF1a↓ →NLRP3、ASC、Caspase-1↓	TLR4/Akt/HIF1a/NLRP3	Wei et al. (2022)
	AD	CORT-induced depressive mice	DHM→AGE、RAGE、IL-1β、IL-6、TNFα↓	NA	Huang et al. (2022)
		APP/PS1 double transgenic AD mice; LPS + ATP induced Mouse microglial BV2 cells	DHM→TLR4、MD2、 IL-1β、IL-6、TNF-α↓	TLR4	Pei et al. (2023)
DHM	Cerebral I/R Injury	Rats of I/R injury; HT22 cells of OGD/R	DMH→GPX4↑, SPHK1、mTOR、p-mTOR、ACSL4、PEBP1↓	SPHK1/mTOR	Xie et al. (2022)
	SAH	SAH rat	DHM→Nrf2、Prx2↑, p-p38、p-ASK1↓	Nrf2	Li et al. (2023)
	Pulmonary Fibrosis	PMLFs; IPF-PHLF	DHM→pSTAT3、GLUT1↓	STAT3/p-STAT3/GLUT1	Li Z et al. (2022)
	Lung Toxicity	Methotrexate-Induced Lung Toxicity Rats	DHM→Nrf2、HO-1、SOD、GSH↑, NF-κB、IL-1β、TGF-β1↓	Nrf2/NF-κB	Matouk et al. (2023)
	ASFV	ASFV induced PAMs	DHM→TLR4、MyD88、p-P38、p-ERK、p-p65、IL-1β、IL-6、IL-18、TNF-α、ROS↓ →GSDMD、GSDMD-N、p30↓	TLR4/MyD88/MAPK/NF-κB	Chen et al. (2023b)
	Obesity	HFD induced obesity	DHM→IRF4、UCP1、PGC-1α↑→promote the browning of WAT	NA	Leng et al. (2022)

Abbreviations: ACSL4, Acyl-CoA, synthetase long-chain family member 4; AD, Alzheimer’s disease; ADMA, asymmetric dimethylarginine; AGE, advanced glycation end products; AKI, acute kidney injury; AKT, Protein kinase B; AMPK, adenosine monophosphate activated protein kinase; Arg1, Arginase-1; ASC, Apoptosis-associated speck-like protein; ASFV, african swine fever virus; ASK1, Apoptosis signal-regulating kinase; Bax, Bcl2-associated X protein; Bcl-2, B-cell lymphoma-2; Bcl-3, B-cell lymphoma-3; BUN, blood urea nitrogen; CAT, catalase; CDCA, chenodeoxycholic acid; CLI, chronic liver injury; CLP, cecum ligation and puncture; CORT, corticosterone; CREB, cAMP-response element binding protein; CS-DHM-NPs, Synthesized chitosan nanoparticles; DDAH1, Dimethylarginine dimethylaminohydrolase-1; DN, diabetic nephropathy; DSS, dextran sulfate sodium; EF, ejection fraction; eNOS, endothelial nitric oxide synthase; ERK, Extracellular signal-regulated kinases; FBG, fasting blood glucose; FINS, fasting serum lisulin; FOXO3a, Forkhead box class O 3a; FS, fractional shortening; FXR, Farnesoid X receptor; GCLC, Glutamate-cysteine ligase, catalytic subunit; CLM, Glutamate-cysteine ligase modifier subunit; GLUT1, Glucose transporter type 1; GPX4, Glutathione peroxidase 4; GSDMD-N, GasderminD-N; GSH-Px, Glutathioneperoxidase; GSK-3β, Glycogen synthase kinase-3β; HbA1c, Hemoglobin A1C; HCD, high cholesterol diet; HFD, High-fat diet; HG, high glucose; HIF-1α, Hypoxia-inducible factor-1α; HO-1, Heme oxygenase-1; HUVEC, human umbilical vein endothelial cell; I/R injury, Ischemia-reperfusion injury; IBD, inflammatory bowel disease; IFN-γ, Interferon-γ; IFP-PHLF, Primary human lung fibroblasts of IPF, patients; IL-10, Interleukin 10; IL-18, Interleukin-18; IL-1β, Interleukin-1β; IL-6, Interleukin 6; IRF4, Interferon regulatory factor 4; IRF4, Interferon regulatory factor 4; JNK, JUN N -terminal kinases; KIM1,Kidney injury molecule-1; LC3-II, Light chain 3-II; LCA, lithocholic acid; LPS, lipopolysaccharide; LPS, lipopolysaccharide; LVEF, left ventricular ejection fraction; LVFS, left ventricular fractional shortening; LVIDd, Left ventricular end-diastolic internal dimension; LVIDs, Left ventricular end-systolic internal dimension; MDA, malondialdehyde; MD2, myeloid differentiation protein; miR-199b-3, MicroRNA-199b-3; miR-21, MicroRNA-21; miR-34a, MicroRNA-4a; miR-5-52p, MicroRNA-5-52p; miR-9, MicroRNA-9; mTOR, mammalian target of rapamycin; MTX, methotrexate; MyD88, Myeloid differentiation factor 88; NA, no applicable; NF-κB, Nuclear factor kappa-B; NLRP3, NOD-like receptor thermal protein domain associated protein 3; NO, nitric oxide; NOS2, Nitric oxide synthase 2; NQO-1, NAPDH: quinone oxidoreductase 1; Nrf2, Nuclear factor erythroid-2-related factor2; OGD/R, Oxygen-glucose deprivation/reperfusion; PAMs, Porcine alveolar macrophages; PDI, protein disulfide isomerase; PDPK1, Phosphoinositide-dependent protein kinase 1; PEBP1, Phosphatidylethanolamine binding protein 1; PGC-1α, Peroxisome proliferator-activated receptor γ coactivator 1α; PI3K, Phosphatidylinositol-3-kinase; PMLFs, Primary mouse lung fibroblasts; Prx2, Peroxiredoxin 2; PTEN, phosphatase and tensin homolog deleted on chromosome ten; RAGE, Receptor for AGE; ROS, reactive oxygen species; SAH, subarachnoid hemorrhages; SIRT1, Sirtuin1; SIRT3,Sirtuin3; SOD, super oxide dismutase; SPHK1, Sphingosine kinase 1; SPHK1, Sphingosine kinases type 1; STAT3, Signal transducer and activator of transcription 3; TAA, thioacetamide; TG, triglyceride; TGF-α, Transforming Growth Factor α; TGR5, G-protein-coupled bile acid receptor; TLR4, Toll-like receptor 4; TNF-α, Tumor necrosis factor-α; UCP1, Uncoupling protein 1; UUO, unilateral uretera obstruction; vWF, von Willebrand factor; WAT, white adipose tissue; α-SMA, α-smooth muscle actin.

### 2.1 Cardiovascular system

#### 2.1.1 Anti-atherosclerosis

In recent years, atherosclerosis (AS) has been characterized as a chronic and progressive inflammatory condition that damages the inner layers of arterial walls, leading to the development of atherosclerotic plaques.

The pathophysiological process of AS is highly complex, with many aspects still needing further exploration. However, the main key points of this process have been generally identified. The early stage of AS development is the “fatty streak” phase, during which low-density lipoprotein (LDL) accumulates in the vascular wall and undergoes oxidation to form oxidized low-density lipoprotein (ox-LDL). This causes damage to the vascular endothelium, inducing the recruitment of monocytes, which mature into M1 pro-inflammatory macrophages (Chistiakov et al., 2017). These macrophages engulf excessive lipids, becoming foam cells, which are characteristic of this stage. As the lesion progresses, more inflammatory cells participate, and vascular smooth muscle cells (VSMCs) and macrophages undergo phenotypic transformation (Vengrenyuk et al., 2015), leading to the formation of atherosclerotic plaques. In the later stages of the lesion, a more stable fibrous cap is formed by a large amount of collagen fibers, smooth muscle cells, a few elastic fibers, and proteoglycans, which results in arterial hardening and narrowing. Several factors can destabilize these plaques, leading to plaque rupture and thrombus formation, potentially triggering cardiovascular events (Poznyak et al., 2020).

Extensive research has demonstrated that DHM produces anti-atherosclerotic effects by reducing oxidative stress and regulating macrophage polarization.

##### 2.1.1.1 Anti-oxidative stress and regulating apoptosis

Recently, the DDAH1-ADMA-eNOS pathway has been recognized as a crucial regulatory mechanism in the production of nitric oxide (NO) and the progression of AS. According to a study by Yang et al. (2020), HM can decrease the expression of microRNA-21 (miR-21) in *Apoe* ^*−/−*^ mice. This microRNA targets dimethylarginine dimethylaminohydrolase-1 (DDAH1), thereby enhancing NO production. This enhancement improves endothelial cell function, reduces vascular inflammation and lipid metabolism disturbances, and lowers the incidence of AS.

Luo et al. (2017) investigated the protective effects and mechanisms of DHM using an ox-LDL-induced human umbilical vein endothelial cell (HUVEC) model. Their research demonstrated that DHM mitigated ox-LDL-induced endothelial cell apoptosis, mitochondrial depolarization, caspase-3 activation, and reactive oxygen species (ROS) production, thus providing cellular protection. Additionally, DHM activated the protein kinase B (Akt) and extracellular signal-regulated kinases 1/2 (ERK1/2) pathways, leading to the stimulation of the Nrf2/HO-1 signaling pathway. This activation promoted the upregulation of antioxidant enzymes and anti-apoptotic proteins, thereby shielding HUVECs from oxidative damage caused by ox-LDL.

Zhang et al. (2019) successfully established an oxidative stress model in HUVECs using sodium nitroprusside (SNP) as a NO donor. Their experimental findings indicate that pre-treatment with DHM reduced intracellular ROS overproduction, decreased malondialdehyde (MDA) levels, and inhibited SNP-induced cell apoptosis in HUVECs. Furthermore, DHM protected HUVECs from oxidative stress by activating the PI3K/Akt/FoxO3a signaling pathway.

##### 2.1.1.2 Regulating macrophage polarization

Macrophages are essential immune cells involved in inflammation and play a crucial role in the development of AS. M1 macrophages promote inflammation, whereas M2 macrophages aid in tissue repair by producing anti-inflammatory factors. Research has shown that miR-9, which is highly expressed in M1 macrophages, promotes M1 polarization by targeting the SIRT1/NF-κB signaling pathway (Wang et al., 2021). Yang et al. (2023) demonstrated that DHM inhibits M1 macrophage polarization and reduces vascular inflammation in AS by suppressing the expression of microRNA-9 (miR-9), potentially through targeting the miR-9/SIRT1/NF-κB signaling pathway in both *in vitro* and *in vivo* studies.

##### 2.1.1.3 Reducing foam cell formation and cholesterol accumulatio

The formation of foam cells and the accumulation of cholesterol are crucial factors in the progression of AS. Sirtuin 3 (SIRT3) has the potential to reduce intracellular ROS levels and inhibit oxidative stress by regulating several mitochondrial enzymes (Wang et al., 2020). Ding et al. (2021) found that the absence of SIRT3 led to increased cholesterol accumulation, oxidative stress, and activation of the NOD-like receptor thermal protein domain-associated protein 3 (NLRP3) in ox-LDL-stimulated macrophages, thereby promoting foam cell formation. This highlights SIRT3 as an effective target for anti-atherosclerosis therapy. Moreover, research indicates that DHM can effectively inhibit this process through a SIRT3-dependent mechanism, demonstrating promising anti-atherosclerosis effects (Ding et al., 2021).

#### 2.1.2 Cardioprotection

Diabetic-induced cardiac damage, known as diabetic cardiomyopathy (DMC), is a significant concern. Studies have shown that DHM can improve cardiac function in diabetic mice by suppressing miR-34a expression and revitalizing impaired autophagy (Ni et al., 2020). Similarly, DHM has been demonstrated to enhance cardiac function in streptozotocin (STZ)-induced diabetic mice (Chen et al., 2023a), ameliorating myocardial hypertrophy, fibrosis, and injury while suppressing oxidative stress, inflammation, and cell death through the activation of SIRT3.

DHM also exhibits cardioprotective effects by mitigating cardiac toxicity. Doxorubicin (DOX), a potent anthracycline antitumor drug, is limited in clinical use due to severe cardiotoxic side effects. Research (Sun et al., 2020) demonstrates that DHM inhibits NLRP3 inflammasome activation through the SIRT1 pathway, effectively preventing DOX-induced cardiac toxicity. *In vitro* and *in vivo* studies conducted by Li X et al. (2022) revealed that DHM protects the heart against DOX-induced toxicity through the activation of the AMPK/mTOR signaling pathway, suppression of apoptosis and oxidative stress, and promotion of protective autophagy.

#### 2.1.3 Prevent platelet activation and thrombosis

DHM exhibits significant potential for antiplatelet effects and thrombus inhibition. Chen et al. (2021) demonstrated that DHM suppresses the expression of platelet p-selectin induced by α-thrombin, thereby inhibiting platelet adhesion by reducing integrin activation and intracellular Ca^2^⁺ elevation during platelet activation. Additionally, DHM may reduce the secretion of von Willebrand factor (vWF) and protein disulfide isomerase (PDI), and impede the activation and expression of endothelial tissue factor (TF), which could prevent thrombus formation. Notably, DHM inhibited platelet aggregation and fibrinogen production without hindering hemostasis.

#### 2.1.4 Reducing vascular calcification

Vascular calcification, a common pathological process in various systemic illnesses, is receiving increasing attention. Studies have shown (Feng et al., 2021) that DHM treatment significantly reduces calcium/phosphate-induced VSMC calcification in rats and humans in a dose-dependent manner. This effect is associated with the inhibition of Akt activation. Notably, DHM’s inhibition of vascular calcification is more potent compared to that of classic Akt inhibitors.

### 2.2 Urinary system

#### 2.2.1 Reducing kidney injury

DHM has been shown to alleviate acute kidney injury (AKI) induced by lipopolysaccharide (LPS) by reducing kidney injury molecule-1 (KIM-1) and blood urea nitrogen (BUN) levels (Wang et al., 2016). Additionally, DHM has been demonstrated to modulate the miR-199b-3p-mediated Nrf2 pathway, thereby attenuating sepsis-induced AKI (Tian et al., 2021).

#### 2.2.2 Inhibiting renal fibrosis

DHM also has significant effects on renal fibrosis. Liu et al. (2019) demonstrated that the abnormal upregulation of miR-34a plays a crucial role in the progression of renal fibrosis, and DHM can effectively treat renal fibrosis by inhibiting miR-34a. Similarly, DHM modulates the miR-155-5p/PTEN signaling pathway and the PI3K/AKT/mTOR signaling pathway in diabetic nephropathy (DN) mice to promote autophagy and mitigate renal interstitial fibrosis (RIF) (Guo et al., 2020b).

#### 2.2.3 Reduce nephrotoxicity

The nephroprotective properties of DHM should not be overlooked. The use of cisplatin as an anti-tumor drug is limited due to its potential renal toxicity. Yan et al. (2023) demonstrated that DHM exerts protective effects against cisplatin-induced AKI by downregulating oxidative stress and inflammatory factors via chitosan nanoparticles loaded with DHM. The study highlights that encapsulated DHM (in a chitosan-based delivery system) more effectively increased Nrf2 levels than the suspension form of DHM, this suggests that encapsulation enhances the bioavailability and antioxidative properties of DHM, allowing it to better activate the Nrf2 pathway and provide stronger protection against oxidative damage.

Another research group also explored the effects of DHM on cisplatin-induced kidney damage (Xu et al., 2023). The study demonstrated that DHM targets the Nrf2/HO-1, mitogen-activated protein kinases (MAPK), and NF-κB signaling pathways to alleviate oxidative stress, inflammation, cell apoptosis, and ferroptosis, thereby exerting a protective effect on the kidneys.

### 2.3 Digestive system

#### 2.3.1 Hepatoprotection

##### 2.3.1.1 Relieving liver injury and hepatotoxicity

Chronic liver injury (CLI) can result from various factors, including drugs and viruses. It serves as a precursor to many serious liver diseases, causing abnormalities in liver metabolic functions and ultimately leading to liver failure. Pyroptosis plays a crucial role in CLI, and research has shown (Cheng et al., 2020) that DHM can reduce the protein expression and mRNA levels of pyroptosis-related molecules, including caspase-1, GasderminD-N (GSDMD-N), and downstream inflammatory molecules, thereby significantly ameliorating carbon tetrachloride (CCl₄)-induced CLI in mice. In addition, another study (Shi et al., 2022) used intraperitoneal injection of LPS in chickens to induce liver injury, followed by treatment with DHM administered by gavage. The results demonstrated that DHM alleviated liver injury by inhibiting LPS-induced activation of the NLRP3 inflammasome and pyroptosis, while also reducing oxidative stress to improve hepatic oxidative homeostasis in chickens. Interestingly, in the assessment of pyroptosis, the study identified GasderminA (GSDMA), an executor protein of pyroptosis, as a key protein involved in LPS-induced pyroptosis in chicken liver injury, and DHM was shown to inhibit GSDMDA, thereby suppressing pyroptosis.

The clinical use of methotrexate (MTX) has been restricted due to its potential hepatotoxicity. However, experimental results with rats by Matouk et al. (2022) indicated that DHM mitigates MTX-induced liver toxicity by reducing oxidative stress and inhibiting the TLR4/NF-κB and NLRP3/caspase-1 pathways. Another team of researchers (Yan et al., 2019) treated L02 cells with emodin to induce hepatotoxicity and then administered DHM to evaluate its protective effects. The study showed that DHM significantly reduced markers of liver cell injury, such as ROS levels and cell apoptosis. Mechanistically, DHM was found to activate the Nrf2 signaling pathway, leading to the upregulation of antioxidant enzymes, including HO-1 and NQO1, which helped to counteract oxidative stress.

Emad et al. (2024) administered DHM to hepatocytes exposed to Valproic acid (VPA) and evaluated its effects on markers of oxidative stress and apoptosis. DHM was found to significantly activate the keap-1/Nrf2/HO-1 pathway, enhancing antioxidant defense mechanisms and reducing oxidative damage. Furthermore, DHM inhibited the NF-κB and caspase-3 pathways, leading to reductions in inflammation and apoptosis. These findings suggest that DHM mitigates VPA-induced hepatotoxicity by activating antioxidant pathways and suppressing inflammation and cell death, underscoring its potential as a protective agent against drug-induced liver injury.

The aforementioned research findings suggest that DHM primarily exerts its hepatoprotective and hepatotoxicity-reducing effects by modulating the Nrf2 pathway and inhibiting pyroptosis.

##### 2.3.1.2 Alleviating alcoholic liver disease

Alcoholic liver disease (ALD) is a growing concern at present. Silva et al. (2020b) conducted research on a mouse model of ethanol (EtOH)-induced ALD and discovered that DHM reduced EtOH-induced hepatic steatosis, oxidative stress, and inflammation both *in vivo* and *ex vivo*. Furthermore, DHM improved ethanol metabolism, resulting in a decrease in ethanol-induced liver damage. It should be noted that the team’s novel use of transperitoneal administration in mice, aimed at increasing the absorption and bioavailability of DHM, yielded unsatisfactory results.

##### 2.3.1.3 Improving non-alcoholic fatty liver disease

The prevalence of non-alcoholic fatty liver disease (NAFLD) is steadily increasing and has become one of the most common chronic liver diseases worldwide. Currently, there are no effective and approved therapeutic drugs for NAFLD. Researchers have summarized the effects and mechanisms of DHM on NAFLD (Gong et al., 2022). DHM exerts its effects primarily through AMPK, NF-κB, and MAPK-related signaling pathways, as well as sirtuin-dependent mechanisms, which align with the existing pathogenesis of NAFLD.

##### 2.3.1.4 Ameliorating hepatic fibrosis and hepatic encephalopathy (HE)

Zhao et al. (2021) demonstrated that treatment with DHM improved liver structure and significantly reduced oxidative stress and hepatotoxicity indices in a mouse model of thioacetamide (TAA)-induced liver fibrosis. Furthermore, DHM reversed TAA-induced liver fibrosis by suppressing inflammation via the PI3K/Akt/NF-κB signaling pathway and apoptosis regulated by transforming growth factor β1 (TGF-β1). Notably, DHM can inhibit the activation of hepatic stellate cells (HSCs) through autophagy induction (Zhou et al., 2021). Additionally, it enhances natural killer cell (NKC)-mediated killing of HSCs by promoting interferon-γ (IFN-γ) secretion, thereby arresting the progression of liver fibrosis.

Additionally, a research team investigated the impact of DHM on hepatic encephalopathy (HE) in a mouse model of acute liver failure induced by thioacetamide (TAA) (Cheng et al., 2021). The findings revealed that DHM restored liver function, improved brain histopathology, and mitigated the symptoms of HE.

#### 2.3.2 Protecting the intestinal barrier function

Inflammatory bowel disease (IBD), which includes ulcerative colitis (UC) and Crohn’s disease (CD), is characterized by chronic inflammation of the intestines, often leading to mucosal ulcers and the eventual degradation of intestinal function (Pineton et al., 2016). Dong et al. (2021) discovered that DHM might alleviate dextran sulfate sodium (DSS)-induced colitis in mouse models by regulating the intestinal microbiota and related bile acid metabolism, while restoring the compromised intestinal barrier function caused by inflammation. The mechanism involves the intestinal microbiota-BAs-FXR/TGR5 signaling pathway.

DHM can also help improve intestinal dysfunction caused by high-intensity exercise (HIE) in mice (Hou et al., 2022), potentially by inhibiting intestinal inflammation and stabilizing intestinal barrier integrity. Additionally, Zhou et al. (2023) investigated the mechanisms behind DHM’s protective effect on the integrity of the intestinal barrier. Their findings reveal that DHM decreases the harm to the intestinal barrier caused by a high-fat diet (HFD) through the stimulation of interleukin 22 (IL-22) expression in group 3 innate lymphoid cells (ILC3). Moreover, this beneficial impact is associated with signal transducer and activator of transcription 3 (STAT3) phosphorylation in the SIRT3 signaling pathway.

### 2.4 Nervous system

#### 2.4.1 Anti-depressant

It is widely recognized that depression can be associated with inflammatory reactions. Research has indicated that DHM has the potential to relieve LPS-induced depressive symptoms and effectively decrease LPS-induced neurotoxicity and inflammatory responses in mice by targeting the TLR4/Akt/HIF-1α/NLRP3 signaling pathway (Wei et al., 2022). Furthermore, a study by Huang et al. (2022) found that DHM alleviated symptoms of chronic depression in corticosterone (CORT)-induced mice. This effect may be attributed to the AGE-RAGE-NF-κB pathway.

#### 2.4.2 Improving memory and cognitive disfunction

Watanabe et al. (2022) conducted a study using a mouse model of social isolation-induced anxiety to assess the effect of DHM. The study determined that administering DHM orally on a daily basis (at a dosage of 2 mg/kg) improved memory and cognition in socially isolated mice, possibly by remodeling hippocampal astrocytes.

Alzheimer’s disease (AD) is characterized by cognitive impairment and is a neurodegenerative condition. Oxidative stress and dysfunction of the cholinergic system are believed to be the primary pathogenic mechanisms. By inhibiting oxidative stress and cholinergic damage, DHM showed significant ameliorative effects on the behavioral and memory deficits induced by D-galactose in aging mice (Sun C et al., 2022). Additionally, DHM targets myeloid differentiation protein 2 (MD2) to inhibit the activation of TLR4 signaling, thereby suppressing neuroinflammation and enhancing cognition in mice with Alzheimer’s disease (Pei et al., 2023).

#### 2.4.3 Treating cerebrovascular diseases

Xie et al. (2022) investigated the protective effect of DHM in a rat model of cerebral ischemia-reperfusion injury. The findings illustrated that DHM hindered ferroptosis by suppressing the SPHK1/mTOR signaling pathway, consequently alleviating cerebral injury due to cerebral ischemia-reperfusion. Furthermore, DHM has been observed to effectively decrease pyroptosis and alleviate ischemic brain injury in rats (Ding et al., 2023).

Similarly, a study explained that DHM is capable of safeguarding the brain via the activation of the Nrf2 and Prx2 signaling pathways, resulting in a notable decline in neuronal oxidative damage and apoptosis after subarachnoid hemorrhage (SAH) (Li et al., 2023). Notably, DHM also diminishes ferroptosis in brain tissue, thereby alleviating cerebral hemorrhages (Liu et al., 2023).

#### 2.4.4 Treating Parkinson’s disease

DHM also has a positive effect on Parkinson’s disease (PD). Guo et al. (2020a) developed a new PD-like mouse model and demonstrated that DHM could alleviate motor dysfunction and prevent the loss of dopaminergic neurons in mice with PD.

#### 2.4.5 Anti-anxiety

DHM improves anxiety through multiple pathways. Studies have revealed that DHM ameliorates anxiety behavior in a chronic social isolation (SI) mouse model by regulating mitochondrial function, reducing oxidative stress, restoring normal autophagy, and increasing brain-derived neurotrophic factor (BDNF), which plays a crucial role in neuroprotection (Al et al., 2022). Additionally, a study conducted by Silva et al. (2020a) on mice demonstrated that DHM could effectively counteract the decrease in adenosine triphosphate (ATP) levels and gephyrin protein expression in the hippocampus due to social isolation, leading to an improvement in anxiety.

### 2.5 Respiratory system

#### 2.5.1 Inhibiting COVID-19 and improving pulmonary fibrosis

In September 2019, the novel coronavirus COVID-19 emerged as a global pandemic. During drug development to target this virus, researchers (Xiao et al., 2021) discovered through molecular docking techniques and *in vitro* cell studies that DHM can effectively suppress SARS-CoV-2 Mpro, thereby inhibiting the novel coronavirus. Moreover, cell research results suggested that DHM might also suppress pulmonary fibrosis and inflammation. Similarly, studies carried out by Li Z et al. (2022) indicated that DHM has the capacity to adjust the STAT3/p-STAT3/GLUT1 signaling pathway, mitigating pulmonary fibrosis induced in a mouse model by bleomycin (BLM). Additionally, these discoveries were confirmed through the use of primary human lung fibroblasts obtained from the lung tissues of idiopathic pulmonary fibrosis (IPF) patients.

#### 2.5.2 Reducing pulmonary toxicity

As previously stated, methotrexate (MTX) is commonly prescribed in clinical settings, but its potential to cause permanent lung damage is a significant hindrance. In their rat study, Matouk et al. (2023) discovered that DHM reduces NF-κB expression in the lung tissue of rats treated with MTX. Furthermore, DHM upregulated the expression of Nrf2/HO-1, thereby alleviating MTX-induced lung toxicity through its antioxidative and anti-inflammatory properties. Interestingly, the study also found that DHM has the potential to reduce the activation of the pro-fibrotic agent TGF-β1 and decrease the weight loss caused by MTX.

### 2.6 Other effects

#### 2.6.1 Anti-virus

Apart from its antiviral effects on the novel coronavirus, DHM also displays antiviral activity against pseudorabies virus (PRV) by inhibiting *in vitro* pyroptosis, regulating the NF-κB signaling pathway, and downregulating apoptosis factors (Zhao et al., 2023; Sun W et al., 2022). Additionally, DHM reduces the levels of inflammatory mediators induced by African swine fever virus (ASFV) by modulating the TLR4/MyD88/MAPK/NF-κB signaling pathway and inhibiting pyroptosis, thus inhibiting ASFV replication (Chen et al., 2023b).

#### 2.6.2 Improving obesity

Currently, the activation of brown adipose tissue (BAT) or induction of browning in white adipose tissue (WAT) has become an increasingly popular target and strategy in the treatment of obesity. Preclinical research conducted by Xiong et al. (2022) has shown that DHM reduces overweight and fat accumulation induced by a high-fat diet in mice and improves glucose and lipid metabolism. Additionally, it promotes the browning of WAT in mice *in vivo*.

Another study in mice produced comparable findings, revealing that DHM promotes the browning of WAT through the activation of the IRF4/PGC-1α pathway (Leng et al., 2022). Furthermore, DHM has been found to regulate bile acid (BA) metabolism and its related effects, including modulating the gut microbiota and the FXR-SREBP-1C-related fat synthesis pathways, which improve obesity (Song et al., 2022).

#### 2.6.3 Anti-tumor

Numerous studies have provided evidence of the potent anti-tumor properties of DHM. A recent review (Wu et al., 2022) summarized the anti-tumor properties of DHM across multiple research domains, including lung cancer, breast cancer, osteosarcoma, ovarian cancer, choriocarcinoma, hepatocellular carcinoma, and gastric cancer.

## 3 How to improve the bioavailability of dihydromyricetin

The aforementioned biological indications clearly demonstrate the regulatory potential of DHM as a natural product, while also highlighting its high safety profile. Currently, the primary obstacle to its further development and utilization is its low bioavailability. Lan et al. (2024) elaborated in detail on the relationship between flavonoid intestinal absorption and bioavailability. Previous studies reported that the oral bioavailability of DHM in rats is only 4.02% (Liu et al., 2017). The main reasons for the low bioavailability of DHM include its low water solubility, poor chemical stability, and low membrane permeability. At 25°C, the solubility of DHM in water is only 0.2 mg/mL (Dong et al., 2023).

In 2019, Liu D et al. (2019) provided a comprehensive summary of methods to enhance the bioavailability of DHM. The main strategies include: increasing DHM’s water solubility (e.g., various nanoparticle systems, cyclodextrin complexes, co-crystallization) and enhancing its lipophilicity (e.g., phospholipid complexes, acylation). Each method has its distinct characteristics.

Recently, several emerging methods have been employed to enhance the bioavailability of DHM, including the preparation of DHM-encapsulated PEGylated liposomes (DHM-Lipo) combined with liposomes (Zhou et al., 2022); an injectable redox albumin-based hydrogel with *in situ* loaded DHM (Deng et al., 2022); and the formulation of chitosan-based nanoparticles loaded with DHM (Yan et al., 2023). Each of these approaches shows promising results. Moreover, the design of ternary complexes (Huang et al., 2023) and the integration of nanoparticle delivery systems with gut microbiota modulation (Lyu et al., 2023) have also been validated in improving the bioavailability of other natural metabolites.

Future research should focus on maximizing the bioavailability of DHM while minimizing any adverse effects on its physicochemical properties. Extracellular vesicles (Feng et al., 2023), as cell-derived microvesicles, have gained significant attention in recent years as a natural drug delivery system due to their robust targeting capabilities, and high biocompatibility. Therefore, combining DHM with extracellular vesicles to improve its bioavailability may be a highly promising research direction.

## 4 Discussion

DHM is a novel natural product with various systemic and health-promoting properties, exhibiting distinctive pharmacological effects. Firstly, DHM’s anti-inflammatory and antioxidant properties stem from its unique chemical structure, making it effective against disorders associated with oxidative stress and inflammation, such as AS and inflammation of the nervous, digestive, and respiratory systems. Secondly, in addition to its impact on numerous signaling pathways associated with inflammation and oxidative stress, DHM regulates various cell death modes, including apoptosis, autophagy, pyroptosis, and ferroptosis. Therefore, cell death-related pathways represent highly promising research directions. Thirdly, DHM has shown synergy with first-line clinical drugs in certain disease treatments, working through multiple pathways and targets. This synergy reduces the severe toxic side effects frequently associated with first-line treatments, such as hepatotoxicity, nephrotoxicity, and cardiotoxicity. DHM’s favorable safety profile enhances tolerance to crucial but highly toxic drugs, increasing its potential for clinical application.

However, current research on DHM remains primarily at the cellular and animal levels, with very few reported clinical studies. This limitation may arise from the instability and relatively low bioavailability of DHM. DHM is soluble only in hot water and ethanol, and its poor solubility in water at room temperature significantly affects its membrane permeability and bioavailability. Furthermore, DHM exhibits inadequate solubility in the gastrointestinal tract, brief circulation time in the bloodstream, poor gastrointestinal absorption, and rapid metabolism (Tong et al., 2015; Zhang et al., 2021). Consequently, researchers are still examining methods to enhance the bioavailability and absorption of DHM, such as the implementation of liposomal carriers, chitosan nanoparticles, and intraperitoneal administration. However, while various strategies to enhance DHM’s bioavailability have been proposed, a thorough comparative analysis of these methods in clinical settings is essential to establish the most effective approach. Additionally, a deeper investigation into the molecular mechanisms by which DHM influences cellular pathways, particularly in relation to inflammation and cell death modalities, could elucidate its therapeutic potential across different diseases.

In conclusion, future research should focus on elucidating DHM’s mechanisms and improving its bioavailability in both preclinical and clinical studies. AS, involving mechanisms such as inflammation, oxidative stress, pyroptosis, and macrophage polarization, aligns well with the pharmacological effects of DHM, making it a promising area for DHM treatment. Additionally, the inflammasome plays an important role in many inflammation-related diseases, and DHM may be a potential inhibitor of the inflammasome. Exploring the effects of DHM on inflammasomes and specific regulatory mechanisms is a meaningful direction. Lastly, understanding the synergistic effects of DHM with existing first-line therapies could lead to more effective treatment regimens, minimizing adverse effects associated with conventional drugs. Expanding the scope of research to include these dimensions will not only strengthen the current understanding of DHM but also pave the way for its practical application in clinical settings. Notably, in 2013, DHM, predominantly found in vine tea, received certification as a ‘new resource food’ from the Chinese Ministry of Health, which has increased researchers’ confidence in endorsing DHM for clinical purposes.

## 5 Criteria for literature selection and search methodology

In this review, we employed a systematic literature retrieval strategy to ensure comprehensive coverage of research on DHM and its pharmacological effects. We first defined our focus, concentrating on the biological activities of DHM and its signaling pathways. To this end, we selected a range of keywords, including “Dihydromyricetin,” “pharmacological effects,” “biological activities,” and “signaling pathways. “For the retrieval logic, we utilized Boolean operators to combine keywords effectively. For instance, we constructed the search query “ (Dihydromyricetin OR DHM) AND (pharmacological effects OR biological activities) AND (signaling pathways OR cell signaling)” to ensure that the search results encompassed all relevant aspects. The literature search was primarily conducted in databases such as PubMed, Web of Science, and Scopus to ensure the acquisition of high-quality academic resources. Through this systematic retrieval strategy, we filtered relevant literature to comprehensively summarize the pharmacological effects and mechanisms of DHM, laying a solid foundation for subsequent discussions and analyses.

## Glossary

ACSL4: Acyl-CoA synthetase long-chain family member 4
AD: Alzheimer’s disease
ADMA: Asymmetric dimethylarginine
AGE: Advanced glycation end product
AKI: Acute Kidney Injury
AKT: Protein kinase B
ALD: Alcoholic liver disease
AMPK: Adenosine monophosphate activated protein kinase
Arg1: Arginase-1
ASC: Apoptosis-associated speck-like protein
ASFV: African swine fever virus
ASK1: Apoptosis signal-regulating kinase
ATP: Adenosine triphosphate
BA: Bile acid
Bax: Bcl2-associated X protein
Bcl-2: B-cell lymphoma-2
Bcl-3: B-cell lymphoma-3
BDNF: Brain-derived neurotrophic factor
BLM: Bleomycin
BUN: Blood urea nitrogen
CAT: Catalase
CCl4: Carbon tetrachloride
CD: Crohn’s disease
CDCA: Chenodeoxycholic acid
CLI: Chronic liver injury
CLP: Cecum ligation and puncture
CORT: Corticosterone
CREB: cAMP-response element binding protein
DDAH1: Dimethylarginine dimethylaminohydrolase-1
DHM: Dihydromyricetin
DMC: Diabetic cardiomyopathy
DN: Diabetic nephropathy
DOX: Doxorubicin
DSS: Dextran sulfate sodium
EF: Ejection fraction
eNOS: Endothelial nitric oxide synthase
ERK1/2: Extracellular signal-regulated kinases 1/2
EtOH: Ethanol
FBG: Fasting blood glucose
FINS: Fasting serum lisulin
FOXO3a: Forkhead box class O 3a
FS: Fractional shortening
FXR: Farnesoid X receptor
GCLC: Glutamate-cysteine ligase, catalytic subunit
GCLM: Glutamate-cysteine ligase modifier subunit
GLUT1: Glucose transporter type 1
GPX4: Glutathione peroxidase 4
GSDMA: GasderminA
GSDMD-N: GasderminD-N
GSH-Px: Glutathioneperoxidase
GSK-3β: Glycogen synthase kinase-3β
HbA1c: Hemoglobin A1C
HCD: High cholesterol diet
HE: Hepatic encephalopathy
HFD: High-fat diet
HG: High glucose
HIE: High-intensity exercise
HIF-1α: Hypoxia-inducible factor-1α
HO-1: Heme oxygenase-1
HSCs: Hepatic stellate cells
HUVEC: Human umbilical vein endothelial cell
I/R injury: Ischemia-reperfusion injury
IBD: Inflammatory bowel disease
IFN-γ: Interferon-γ
IFP-PHLF: Primary human lung fibroblasts of IPF patients
IL-10: Interleukin 10
IL-18: Interleukin-18
IL-1β: Interleukin-1β
IL-22: Interleukin 22
IL-6: Interleukin 6
ILC3s: Group 3 innate lymphoid cells
IRF4: Interferon regulatory factor 4
JNK: JUN N -terminal kinases
KIM1: Kidney injury molecule-1
LC3-II: Light chain 3-II
LCA: Lithocholic acid
LPS: Lipopolysaccharide
LVEF: Left ventricular ejection fraction
LVFS: Left ventricular fractional shortening
LVIDd: Left ventricular end-diastolic internal dimension
LVIDs: Left ventricular end-systolic internal dimension
MDA: Malondialdehyde
MAPK: Mitogen-activated protein kinases
MD2: Myeloid differentiation Protein
miR-155-5p: MicroRNA-155-5p
miR-199b-3: MicroRNA-199b-3
miR-21: MicroRNA-21
miR-34a: MicroRNA-4a
miR-5-52p: MicroRNA-5-52p
miR-9: MicroRNA-9
mTOR: Mammalian target of rapamycin
MTX: Methotrexate
MyD88: Myeloid differentiation factor 88
NA: No applicable
NAFLD: Non-alcoholic fatty liver disease
NF-κB: Nuclear factor kappa-B
NKC: Natural killer cell
NLRP3: NOD-like receptor thermal protein domain associated protein 3
NO: Nitric oxide
NOS2: Nitric oxide synthase 2
NQO-1: NAPDH: quinone oxidoreductase 1
Nrf2: Nuclear factor erythroid-2-related factor2
OGD/R: Oxygen-glucose deprivation/reperfusion
Ox-LDL: Oxidized low-density lipoprotein
PAMs: Porcine alveolar macrophages
PD: Parkinson’s disease
PDI: Protein disulfide isomerase
PDPK1: Phosphoinositide-dependent protein kinase 1
PEBP1: Phosphatidylethanolamine binding protein 1
PGC-1α: Peroxisome proliferator-activated receptor γ coactivator 1α
PI3K: Phosphatidylinositol-3-kinase
PMLFs: Primary mouse lung fibroblasts
PRV: Pseudorabies virus
Prx2: Peroxiredoxin 2
PTEN: Phosphatase and tensin homolog deleted on chromosome ten
RAGE: Receptor for AGE
RIF: Renal interstitial fibrosis
ROS: Reactive Oxygen Species
SAH: Subarachnoid hemorrhages
SI: Social isolation
SIRT1: Sirtuin1
SIRT3: Sirtuin3
SNP: Sodium nitroprusside
SOD: Super oxide dismutase
SPHK1: Sphingosine kinases type 1
SREBP-1C: Sterol regulatory element binding protein-1C
STAT3: Signal transducer and activator of transcription 3
STZ: Streptozotocin
TAA: Thioacetamide
TF: Tissue factor
TG: Triglyceride
TGF-α: Transforming growth factor α
TGF-β1: Transforming growth factor β1
TGR5: G-protein-coupled bile acid receptor
TLR4: Toll-like receptor 4
TNF-α: Tumor necrosis factor-α
UC: Ulcerative colitis
UCP1: Uncoupling protein 1
UUO: Unilateral uretera obstruction
VSMC: Vascular smooth muscle cell
vWF: von Willebrand factor
WAT: White adipose tissue
α-SMA: α-smooth muscle actin
